# Population-Based Hospitalization Burden of Influenza A Virus Subtypes and Antigenic Drift Variants in Children in Hong Kong (2004–2011)

**DOI:** 10.1371/journal.pone.0092914

**Published:** 2014-04-30

**Authors:** Susan S. Chiu, Janice Y. C. Lo, Kwok-Hung Chan, Eunice L. Y. Chan, Lok-Yee So, Peng Wu, Benjamin J. Cowling, Robin Chen, J. S. Malik Peiris

**Affiliations:** 1 Department of Pediatrics & Adolescent Medicine, The University of Hong Kong, Pokfulam, Hong Kong; 2 Public Health Laboratory Services Branch, Centre for Health Protection, Department of Health, Kowloon, Hong Kong; 3 Department of Microbiology, The University of Hong Kong, Pokfulam, Hong Kong; 4 Department of Pediatrics and Adolescent Medicine, Pamela Youde Nethersole Eastern Hospital, Chai Wan, Hong Kong; 5 Division of Epidemiology and Biostatistics, School of Public Health, The University of Hong Kong, Pokfulam, Hong Kong; 6 Centre of Influenza Research, School of Public Health, The University of Hong Kong, Pokfulam, Hong Kong; The University of Chicago, United States of America

## Abstract

**Objectives:**

We aim to document and analyze influenza hospitalization burden in light of antigenic changes in circulating influenza viruses in Hong Kong.

**Methods:**

The pediatric age-specific rates of influenza A hospitalization in Hong Kong for 2004–2011 which encompassed the emergence of H1N1pdm09 were extrapolated from admissions to 2 hospitals that together catered for 72.5% of all pediatric admissions on Hong Kong Island. Influenza A was detected by immunofluorescence, culture and/or PCR on nasopharyngeal aspirates.

**Results:**

Influenza A caused high rates of hospitalization in children with year to year fluctuations. The highest hospitalization burden was seen with H1N1pdm09 in 2009. Additional factors affecting hospitalization were the proportion of viral circulation among different subtypes, and antigenic drifts. Taking these into effect, an H3N2 dominated year was not always associated with more hospitalizations than a ‘seasonal’ H1N1 year. Hospitalization burden was higher in seasons when drifted viruses of H1N1 or H3N2 dominated. No hospitalization was documented in infants <6 months of age during years when an undrifted virus circulated (2006 for H1N1 and 2008 for H3N2) but significant hospitalization was observed with a drifted or shifted virus (2004, 2005, 2007 and 2010 for H3N2, and 2009 for H1N1pdm09).

**Conclusions:**

We documented a consistently high pediatric hospitalization burden of influenza A. Knowledge of antigenic changes and their proportion of circulation aids in the interpretation of impact of the subtypes. Year-to-year variation in hospitalization rates in young infants appeared to correlate with antigenic variation, lending support to the role of protection from maternal antibodies.

## Introduction

Influenza leads to a significant burden of hospitalization in children annually. In recent years, improved surveillance efforts and modeling have produced an increasing number of documentation on the impact of influenza on hospitalization [1]–[9]. However, influenza disease and hospitalization are the outcome of dynamic interaction between the virus and the susceptible hosts in the population. In order to truly gain insight into the impact of influenza hospitalization, the data should be interpreted in light of the subtypes and antigenic drifts and even shift. These data are lacking in the literature. In this study, we seek to compare the age-specific hospitalization disease burden of influenza A in children in Hong Kong over an 8 year period that includes 5 years of interpandemic influenza epidemics, the first pandemic wave in 2009 and two subsequent influenza epidemics, to examine the impact of influenza A subtypes, drift variants and pandemics on population based hospitalization rates in children. We specifically examined the impact of antigenic changes on infants under 6 months of age since we hypothesized that they would be protected by maternal antibodies in the absence of antigenic change.

## Materials and Methods

The Hong Kong Special Administrative Region (SAR) of China is made up of Hong Kong Island, Kowloon peninsular, the New Territories and some sparsely populated outlying islands. In 2006, there were 195,922 persons <18 years residing in Hong Kong Island [10]. Pamela Youde Nethersole Eastern Hospital (PYNEH) and Queen Mary Hospital (QMH) were the only 2 acute public hospitals on Hong Kong Island serving this population. These two hospitals, with a total of 153 pediatric beds, catered for 72.5% of all pediatric admissions from the population of Hong Kong Island in 2006 [11] (the remainder being admitted to private hospitals). While Hong Kong Island is not an entirely closed community, parents from other parts of Hong Kong SAR, namely Kowloon and the New Territories, rarely bring their children across the harbor for acute general pediatric problems since there are 10 public hospitals with pediatric inpatient service serving those areas. Thus we can relate admission to PYNEH and QMH to the population resident on Hong Kong Island to derive population based estimates of disease burden. Census data were used to define the age-stratified population at risk.

Acute respiratory infection was defined as fever ≥38°C with any respiratory symptom including cough, runny nose, sore throat. Patients aged <18 years with a Hong Kong Island home address admitted were recruited on two 24-hour periods of the week, 1 day each from PYNEH and QMH. All recruited patients had an NPA sent for virological testing. The study period included in this report was from January 2004 to December 2011. The data from 2004–2006 have been previously published [10]. The inclusion of a longer time-series, and the virus subtype and strain data from the virology laboratory of the Department of Health, Hong Kong SAR now allows a more detailed analysis of the impact of seasonal and pandemic virus subtypes and antigenic drift variants on influenza hospitalization disease burden.

### Ethics Statement

The study protocol was approved by the joint Institutional Review Board of the University of Hong Kong and Queen Mary Hospital (Hong Kong) which waived the written consent since the investigation was a routine diagnostic test carried out as part of routine care, and any patient information was delinked from individual patient identification to maintain patient confidentiality.

### Virology testing of hospitalized patients

NPA specimens were tested for influenza A by direct antigen detection by direct immunofluorescence (IF) and virus culture in all recruited patients at the Virology Laboratory at the University of Hong Kong. The direct immunofluorescence antigen test was carried out as previously described using IMAGEN™ respiratory screen and typing reagents (Oxoid Ely Ltd., UK) [12]. All the specimens found positive in the respiratory screen with a pooled IF reagent were further identified using antibody reagents to the individual virus (influenza A or B, respiratory syncytial virus, parainfluenza virus type 1, 2, 3 and adenovirus) using the IMAGEN™ typing kit.

To culture the respiratory viruses, MDCK, LLC-MK2, HEp-2C and RD cell monolayers in culture tubes were inoculated with 150 µl of the nasopharyngeal aspirate-virus transport medium suspension and the virus isolation was carried out as previously described [13]. MDCK is the cell line for influenza isolation, and inoculated tubes were maintained in serum free medium with trypsin (2 µg/ml), and incubated at 33°C for 7 days. The cultures were examined daily for cytopathic effect and IF and hemagglutination inhibition tests were used for identification and antigenic characterization of influenza viruses respectively.

In addition, during the pandemic wave in 2009, the NPA samples were tested for influenza A H1N1pdm09 by RT-PCR. Total nucleic acid from 250 µl NPA was extracted by using NucliSens easyMAG instrument (bioMerieux, Netherlands) according to the manufacturer's instruction and previously described. Nucleic acid was recovered in 55 µl elution buffer and was kept at −80°C until use. The diagnosis of influenza A virus was performed by RT-PCR targeting the M gene as previously described [14]–[16]. Real-time one-step RT-PCR assays were used for the detection of pandemic A (H1N1) virus using Invitrogen SuperScript III Platinum One-Step Quantitative Kit in a 7500 Sequence Detection System (Applied Biosystem, Foster City, CA, USA) [17]. Briefly, 5 µl of eluted RNA was amplified in a 25 µl reaction containing 0.5 µl Superscript III Reverse Transcriptase/Platinum Taq DNA polymerase (Invitrogen), 0.05 µl ROX reference dye (25 µM), 12.5 µl of 2X reaction buffer, 800 nmol/l each of forward primer (5′-CCAAAGCTCAGCAAATCCTACAT-3′), reverse primer (5′-GATGGTGAATGCCCCATAGC-3′), and probe 200 nmol/l (Fam-TGATAAAGGGAAAGAAGTCCT-MGB), Reactions were first incubated at 50°C for 30 min, followed by 95°C for 2 min, and were then thermal cycled for 50 cycles (95°C for 15 s, 55°C for 30 s).

In addition, the virology laboratory of the Department of Health provides diagnostic service for influenza virus detection for the whole territory of Hong Kong with approximately 95% of the positive results detected from specimens from adult and pediatric patients hospitalized in public and private hospitals, the rest from out-patient clinics. Virus strain typing is performed on all influenza virus isolates. As the National Influenza Centre within the World Health Organization (WHO) influenza laboratory network, strain surveillance data are recorded for regular contribution to the WHO Global Influenza Programme.

### Statistical Analysis

Hospitalization rates (subtype-specific annual hospitalization rates and monthly hospitalization rates) and 95% confidence intervals for each age group were estimated based on the Poisson distribution. The numerator in each incidence rate was the number of laboratory-confirmed admissions in that age group, while the denominator was the estimated person-years at risk each year. The latter was estimated by the population of Hong Kong Island in that age group in 2006, divided by 7 to allow for the study design of sampling 1 day per week, and further divided by 0.725 to allow for the proportion of children served in the 2 public hospitals. Census data for infants under 6 months were lacking. Therefore, we used half the population under 1 year of age as the denominator, assuming a constant birth rate over the year. All statistical analyses were conducted using SAS version 9.02 and R version 2.15.1 (R Foundation for Statistical Computing, Vienna, Austria).

## Results

In this study, 89% of Influenza A was detected by both direct immunofluorescence (IF) and virus culture, and another 11% was detected by virus culture alone. All the samples were tested by PCR only during the H1N1 pandemic period. The circulation of influenza A strains in Hong Kong during the study years as well as information dating back to 2003 is shown (Table 1). Circulation data from 2003 are included to provide background for the following years. H3N2 predominated in 4 out of the 8 years and co-circulated with H1N1 in 2008. The first case of H1N1pdm09 in Hong Kong was diagnosed on 1 May 2009 in a traveler from Mexico arriving in Hong Kong [18]. From then until the end of June 2009, Hong Kong implemented a containment strategy where all patients confirmed to be infected with H1N1pdm09 were hospitalized and discharged according to isolation and infection control measures, regardless of clinical indication. Starting June 29, 2009, when community transmission of H1N1pdm09 was widespread, the Department of Health and the Hospital Authority of Hong Kong switched to mitigation measures, and adjusted the policy to hospitalize on purely clinical basis [18].

**Table 1:** Circulation of influenza A strains reflected by proportion of influenza isolates submitted to the virology laboratory of the Department of Health in Hong Kong (2003–2011).

Year	Number of Isolates	H1N1		H3N2	
		Strains	No. of isolates (%)	Strains	No. of isolates (%)
2003	1428	A/New Caledonia/20/99(H1N1)-like	9 (0.6%)	**A/Moscow/10/99(H3N2)-like**	**1419 (99.4%)**
2004	4542	A/New Caledonia/20/99(H1N1)-like	26 (0.57%)	**A/Fujian/411/2002(H3N2)-like**	**4561 (99.4%)**
2005	4692	A/New Caledonia/20/99(H1N1)-like	410 (8.7%)	**A/California/7/2004(H3N2)-like**	**4282 (91.3%)**
2006	3090	A/New Caledonia/20/99(H1N1)-like	2880 (93.2%)	A/California/7/2004(H3N2)-like	210 (6.8%)
2007	4742	A/New Caledonia/20/99(H1N1)-like	127 (2.6%)	**A/Wisconsin/67/2005(H3N2)-like**	**4615 (97.4%)**
2008	3237	**A/Solomon Islands/3/2006(H1N1)-like**	**1677 (51.8%)**	A/Brisbane/10/2007(H3N2)-like	1560 (48.2%)
		**A/Brisbane/59/2007(H1N1)-like**			
2009	36289	**A/Brisbane/59/2007(H1N1)-like**	**2855 (8.0%)**	**A/Perth/16/2009(H3N2)-like**	**7252 (20.2%)**
		**A/California/7/2009(H1N1)-like**	**25633 (71.7%)**		
2010	7138	A/California/7/2009(H1N1)-like	2874 (40.3%)	A/Perth/16/2009(H3N2)-like	4264 (59.7%)
2011	5085	A/California/7/2009(H1N1)-like	3782 (74.4%)	A/Victoria/361/2011(H3N2)-like	1303 (25.6%)

Strains with decreased homology from the previous year are denoted in bold.

### Hospitalization disease burden

Pediatric hospitalization disease burden projected for the whole of Hong Kong remained high over the 8 study years but with year-to-year fluctuation (Table 2). Information on mortality and intensive care admission was not captured in this study. Influenza vaccination data in the recruited children within 6 months of hospitalization was available for 2009–2011: 17.2% in 2009, 12.1% in 2010 and 9.6% in 2011. Total pediatric hospitalization for H1N1pdm09 in 2009 after the containment period was 2 to 3 times that of seasonal influenza A in preceding years. In general, hospitalization burden was higher in years when antigenic changes appeared. Despite common belief that H3 causes more severe infection than H1, it is not apparent from our study that this is the case for hospitalization in children. For example, in 2008 when a drifted H1N1 and a non-drifted H3N2 were identified in approximately equal proportions, H1N1 caused more than twice the hospitalizations caused by H3N2.

**Table 2:** Annual pediatric hospitalizations due to influenza A strains in children aged ≤18 y in Hong Kong in comparison with the proportion of positive isolates at the Virology Laboratory of the Department of Health of Hong Kong.

Year	Overall	H1N1		H3N2		H1N1pdm09	
	Hospitalization	Hospitalization (%)	Isolation Proportion (%)	Hospitalization (%)	Isolation Proportion (%)	Hospitalization (%)	Isolation Proportion (%)
**2004**	2045	0	0.6%	**2045 (100%)**	**99.4%**	0	-
**2005**	2053¶	400 (19.5%)	8.7%	**1599 (77.9%)**	**91.3%**	0	-
**2006**	1562	1499 (96.0%)	93.7%	63 (4.0%)	6.8%	0	-
**2007**	2226¶	176 (7.9%)	2.6%	**1994 (89.5%)**	**97.4%**	0	-
**2008**	1940	**1331 (68.6%)**	**51.8%**	609 (31.4%)	48.2%	0	-
**2009**	5832	**1115 (19.1%)**	**8.0%**	**1164 (19.9%)**	**20.0%**	**3553 (61.0%)**	**71.7%**
						**3202** * **(54.9%)**	
**2010**	2112	0	0	1174 (55.6%)	59.7%	938 (44.4%)	40.3%
**2011**	1943	0	0	624 (32.1%)	25.6%	1319 (67.9%)	74.4%

Influenza strain drifts and shift (in 2009) are denoted in bold.

¶ No subtype available in 2 patients.

* Admissions associated with H1N1pdm09 during the containment period were excluded.

Between 8 and 25% of the hospitalized children had one or more documented underlying condition putting them at risk for influenza complications. (Table 3) Hospitalization rates in infants under 6 months of age were of particular interest. There were high hospitalization rates for this age group in 2004 and 2005, the years predominated by a drifted A/Fujian/411/02 (H3N2)-like virus and A/California/7/2004 (H3N2)-like virus, respectively. (Table 4) There was no hospitalization in infants under 6 months of age in 2006 when A/New Caledonia/20/99 H1N1-like virus which had been in circulation since 1999 predominated. A drifted A/Wisconsin/67/2005 (H3N2)-like virus predominated in 2007, again causing significant hospitalization in the youngest age groups. 2008 was a year with mixed circulation involving 2 drifted H1N1 strains (A/Brisbane/59/2007 and to a small extent A/Solomon Islands/3/2006) as well as an undrifted H3N2 (A/Brisbane/10/2007). There was significant hospitalization for H1N1 but not for H3N2 in this youngest age group.

**Table 3:** Age-specific rates (95% CI) of hospitalizations associated with confirmed influenza A per 10,000 residents, 2004–2011.

Year	Proportion of hospitalized children with ≥1 underlying condition	<6 m	6–12 m	<1 y	1–<2 y	2–<5 y	5–<10 y	10–<15 y	15–<18 y
2004	13.5%	25.9	129.7	77.8	95.5	63.5	10.9	2.9	2.4
		(0.7, 144.5)	(42.1, 302.6)	(28.5, 169.3)	(38.4, 196.7)	(35.5, 104.7)	(4.0, 23.7)	(0.4, 10.6)	(0.1, 13.6)
2005	8.1%	129.7	77.8	103.7	40.9	42.3¶	21.8	2.9	4.9
		(42.1, 302.6)	(16.0, 227.3)	(44.8, 204.4)	(8.4, 119.6)	(20.3, 77.8)	(11.3, 38.1)	(0.4, 10.6)	(0.6, 17.6)
2006	25%	0.0	77.8	38.9	54.6	38.1	18.2	1.5	2.4
		(0.0, 95.7)	(16.0, 227.3)	(8.0, 113.7)	(14.9, 139.7)	(17.4, 72.3)	(8.7, 33.4)	(0.0, 8.2)	(0.1, 13.6)
2007	17.5%	51.9	77.8	64.8	68.2	80.4¶	14.5	2.9	2.4
		(6.3, 187.3)	(16.0, 227.3)	(21.0, 151.3)	(22.1, 159.2)	(48.4, 125.5)	(6.3, 28.7)	(0.4, 10.6)	(0.1, 13.6)
2008	11.4%	51.9	77.8	64.8	68.2	76.2	7.3	4.4	0.0
		(6.3, 187.3)	(16.0, 227.3)	(21.0, 151.3)	(22.1, 159.2)	(45.1, 120.4)	(2.0, 18.6)	(0.9, 12.9)	(0.0, 9.0)
2009	16.3%	155.6	155.6	155.6	218.3	122.7	52.7	17.6	14.6
		(57.1, 338.6)	(57.1, 338.6)	(80.4, 271.8)	(124.8, 354.4)	(82.2, 176.2)	(35.3, 75.7)	(9.1, 30.7)	(5.4, 31.8)
2010	7.9%	103.7	0.0	51.9	68.2	84.6	12.7	2.9	0.0
		(28.3, 265.6)	(0.0, 95.7)	(14.1, 132.8)	(22.1, 159.2)	(51.7, 130.7)	(5.1, 26.2)	(0.4, 10.6)	(0.0, 9.0)
2011	22.9%	103.7	77.8	90.8	54.6	59.2	10.9	2.9	4.9
		(28.3, 265.6)	(16.0, 227.3)	(36.5, 187.0)	(14.9, 139.7)	(32.4, 99.4)	(4.0, 23.7)	(0.4. 10.6)	(0.6, 17.6)

¶ No subtype data were available for 2 patients.

**Table 4:** Age-specific rates (95% CI) of hospitalization for different influenza A subtypes per 10,000 residents 2004–2011.

Type/subtype	Age	2004	2005	2006	2007	2008	2009	2010	2011
H1N1	<6 m	0.0 (0.0, 95.7)	0.0 (0.0, 95.7)	0.0 (0.0, 95.7)	0.0 (0.0, 95.7)	51.9 (6.3, 187.3)	51.9 (6.3, 187.3)	-	-
	6–12 m	0.0 (0.0, 95.7)	25.9 (0.7, 144.5)	77.8 (16.0, 227.3)	0.0 (0.0, 95.7)	25.9 (0.7, 144.5)	0.0 (0.0, 95.7)	-	-
	<1 y	0.0 (0.0, 47.8)	13.0 (0.3, 72.2)	38.9 (8.0, 113.7)	0.0 (0.0, 47.8)	38.9 (8.0, 113.7)	25.9 (3.1, 93.7)	-	-
	1–<2 y	0.0 (0.0, 50.3)	0.0 (0.0, 50.3)	54.6 (14.9, 139.7)	0.0 (0.0, 50.3)	54.6 (14.9, 139.7)	27.3 (3.3, 98.6)	-	-
	2–<5 y	0.0 (0.0, 15.6)	4.2 (0.1, 23.6)	38.1 (17.4, 72.3)	0.0 (0.0, 15.6)	50.8 (26.2, 88.7)	33.9 (14.6, 66.7)	-	-
	5–<10 y	0.0 (0.0, 6.7)	5.5 (1.1, 15.9)	18.2 (8.7, 33.4)	3.6 (0.4, 13.1)	5.5 (1.1, 15.9)	12.7 (5.1, 26.2)	-	-
	10–<15 y	0.0 (0.0,5.4)	1.5 (0.0, 8.2)	1.5 (0.0, 8.2)	0.0 (0.0,5.4)	2.9 (0.4, 10.6)	1.5 (0.0, 8.2)	-	-
	15–<18 y	0.0 (0.0, 9.0)	2.4 (0.1, 13.6)	0.0 (0.0, 9.0)	2.4 (0.1, 13.6)	0.0 (0.0, 9.0)	0.0 (0.0, 9.0)	-	-
H3N2	<6 m	25.9 (0.7, 144.5)	129.7 (42.1, 302.6)	0.0 (0.0, 95.7)	51.9 (6.3, 187.3)	0.0 (0.0, 95.7)	25.9 (0.7, 144.5)	51.9 (6.3, 187.3)	0.0 (0.0, 95.7)
	6–12 m	129.7 (42.1, 302.6)	51.9 (6.3, 187.3)	0.0 (0.0, 95.7)	77.8 (16.0, 227.3)	51.9 (6.3, 187.3)	25.9 (0.7, 144.5)	0.0 (0.0, 95.7)	0.0 (0.0, 95.7)
	<1 y	77.8 (28.5, 169.3)	90.8 (36.5, 187.0)	0.0 (0.0, 47.8)	64.8 (21.0, 151.3)	25.9 (3.1, 93.7)	25.9 (3.1, 93.7)	25.9 (3.1, 93.7)	0.0 (0.0, 47.8)
	1–<2 y	95.5 (38.4, 196.7)	40.9 (8.4, 119.6)	0.0 (0.0, 50.3)	68.2 (22.1, 159.2)	13.6 (0.3, 76.0)	54.6 (14.9, 139.7)	27.3 (3.3, 98.6)	13.6 (0.3, 76.0)
	2–<5 y	63.5 (35.5, 104.7)	33.9 (14.6, 66.7)	0.0 (0.0, 15.6)	76.2 (45.1, 120.4)	25.4 (9.3, 55.3)	46.5 (23.2, 83.3)	46.5 (23.2, 83.3)	25.4 (9.3, 55.3)
	5–<10 y	10.9 (4.0, 23.7)	16.4 (7.5, 31.1)	0.0 (0.0, 6.7)	10.9 (4.0, 23.7)	1.8 (0.0, 10.1)	5.5 (1.1, 15.9)	7.3 (2.0, 18.6)	5.5 (1.1, 15.9)
	10–<15 y	2.9 (0.4, 10.6)	1.5 (0.0, 8.2)	0.0 (0.0,5.4)	2.9 (0.4, 10.6)	1.5 (0.0, 8.2)	1.5 (0.0, 8.2)	2.9 (0.4, 10.6)	0.0 (0.0, 5.4)
	15–<18 y	2.4 (0.1, 13.6)	2.4 (0.1, 13.6)	2.4 (0.1, 13.6)	0.0 (0.0, 9.0)	0.0 (0.0, 9.0)	0.0 (0.0, 9.0)	0.0 (0.0, 9.0)	2.4 (0.1, 13.6)
H1N1pdm09	<6 m	-	-	-	-	-	77.8 (16.0, 227.3)	51.9 (6.3, 187.3)	103.7 (28.3, 265.6)
	6–12 m	-	-	-	-	-	129.7 (42.1, 302.6)	0.0 (0.0, 95.7)	77.8 (16.0, 227.3)
	<1 y	-	-	-	-	-	103.7 (44.8, 204.4)	25.9 (3.1, 93.7)	90.8 (36.5, 187.0)
	1–<2 y	-	-	-	-	-	136.4 (65.4, 250.9)	40.9 (8.4, 119.6)	40.9 (8.4, 119.6)
	2–<5 y	-	-	-	-	-	42.3 (20.3, 77.8)	38.1 (17.4, 72.3)	33.9 (14.6, 66.7)
	5–<10 y	-	-	-	-	-	34.6 (20.8, 54.0)	5.5 (1.1, 15.9)	5.5 (1.1, 15.9)
	10–<15 y	-	-	-	-	-	14.7 (7.0, 27.0)	0.0 (0.0, 5.4)	2.9 (0.4, 10.6)
	15–<18 y	-	-	-	-	-	14.6 (5.4, 31.8)	0.0 (0.0, 9.0)	2.4 (0.1, 13.6)

- There was no circulation of ‘seasonal’ influenza A(H1N1) after 2010, and no circulation of influenza A(H1N1pdm09) before 2009 in Hong Kong.

Overall influenza hospitalization rates in 2009 were high in all pediatric age groups compared to the previous years. The pattern of hospitalization for H1N1pdm09 was different from that of seasonal influenza: while hospitalization rates were highest amongst young children <5 years in seasonal influenza seasons, significant hospitalization was seen amongst school age children for H1N1pdm09 between 2 to <10 years and even in adolescents 10 to <18 years. By 2010 and 2011, the overall burden of influenza declined to the magnitude of seasonal influenza seen in previous years (Table 1).

### Annual hospitalization pattern for influenza A (2007–2011)

**Figure 1:**
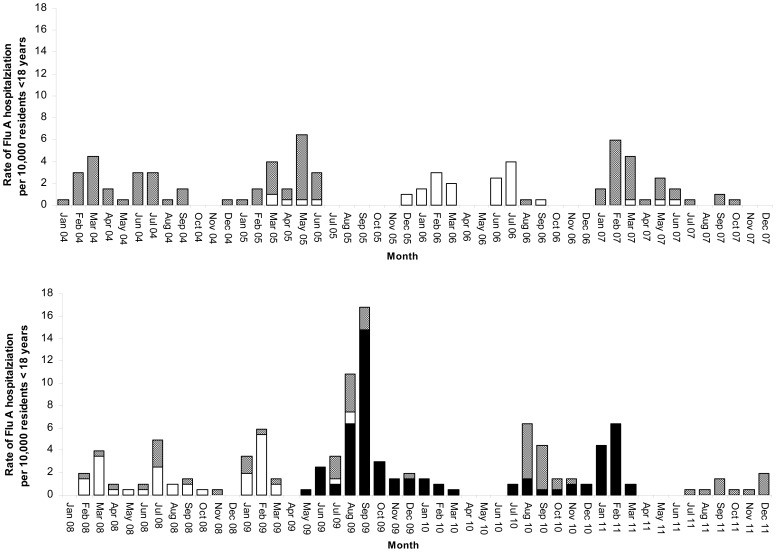
Rate of influenza A hospitalizations per 10,000 residents <18 years: influenza A(H3N2) (gray); influenza A(H1N1) (white); influenza A(H1N1)pdm09 (black).

Hospitalization rates by month from 2004 to 2011 showed that the influenza season in Hong Kong was quite unpredictable. (Figure 1A and 1B) Peak winter influenza hospitalization happened in January and February in only 2 years (2009 and 2011) and in the later months of February and March in 4 years (2004, 2006, 2007, 2008). A second peak was seen in 6 years with varying magnitudes from year to year.

**Figure 2:**
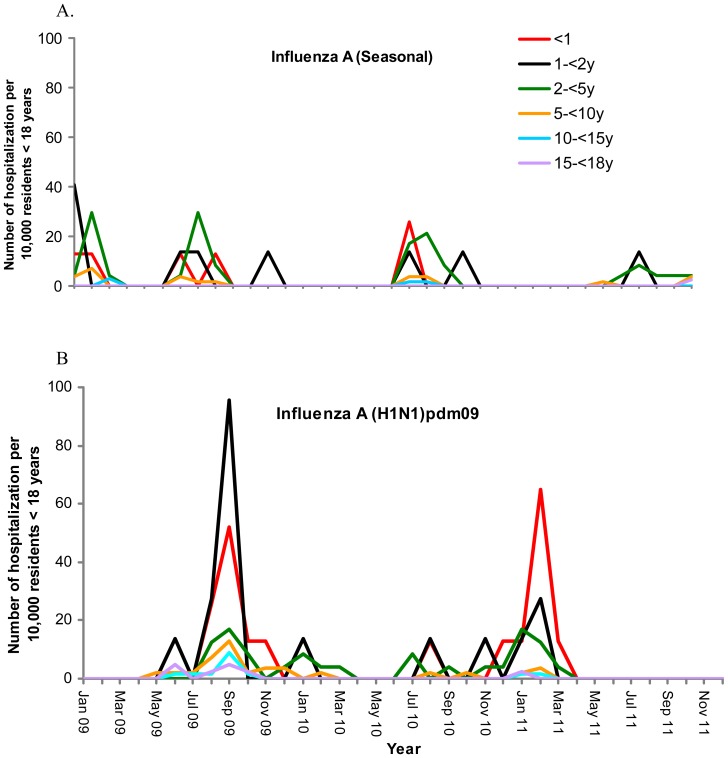
A & B. Age-specific hospitalization rates for seasonal influenza A and H1N1pdm09 (2009–2011).

The first known imported case of H1N1pdm09 arrived in Hong Kong on 30 April 2009 and was detected on 1 May 2009 but significant pediatric hospitalization was not documented until June. The very first cases in May were imported cases and a bit later in early June, cases were students attending international schools or children with western contacts. (Figure 2) By August, hospitalization was mainly in children <5 years who acquired their infection locally. During the H1N1pdm09 peak, young children were also hospitalized for H3N2 and seasonal H1N1 at significant rates. In fact, the hospitalization rate due to seasonal influenza in 2–<5 year olds in August 2009 was more than double the hospitalization rate for H1N1pdm09 in that age group. Hospitalization rates in the winter influenza season of 2010–2011 were in the young age group as usually seen with seasonal influenza.

## Discussion

We continue to document high hospitalization due to influenza A in children at a population level in Hong Kong. A recent US study documenting the influenza burden in children <5 years of age from 2004 to the season before the 2009 pandemic showed similar high hospitalization rates for influenza A in infants <6 months, but 3- or 4- fold lower than what we document here [19]. The difference in the 2–<5 years group was even higher with a 10-fold difference. Pandemic H1N1 resulted in high rates of hospitalization in young children to the extent that was not seen in the prepandemic years, as well as hospitalization in much older children and adolescents that was unusual. In serologic studies, we estimated that approximately half of all local children 5–14 years were infected with H1N1pdm09 by December 2009 [20]–[22]. Hospitalization was highest in the young children, in keeping with findings around the world although our hospitalization rates were consistently higher than that reported in most other places [10], [23]–[29]. Different methodologies were used in these reports. Most were retrospective in nature and some relied on passive surveillance to different degrees, which could suffer from under-recognition and under-testing. It is unclear if the difference in hospitalization rates was a result of low influenza vaccination rates in Hong Kong children. This study did not examine severity but there was no intensive care admission in our study population and a previous study of children hospitalized for H1N1pdm09 and seasonal H3N2 and H1N1 had rather mild diseases [30]. Different hospitalization thresholds therefore could also likely partially explain the difference seen. Yet another potential explanation to the higher hospitalization rates seen in Hong Kong may be this reflects a higher infection rate in a crowded place like Hong Kong.

Regardless of the absolute hospitalization rates, the trends we documented were similar to that reported elsewhere. The impact in disease burden of this pandemic was much less than initially feared, mounting to only 2- to 3-fold that of seasonal influenza documented in the previous 5 years. (Table 2) In Argentina, the pandemic influenza also only resulted in a doubling of hospitalization in children compared to 2008 [27]. Like us, investigators in the Netherlands also reported that the 5 to 14 year olds had the highest relative increase in influenza-like illness during the pandemic compared to previous years while documenting the highest hospitalization rate in younger children [26].

Hospitalization burden is the composite result of H3 vs. H1 influenza strain, the novelty of the influenza strain and therefore population at risk, and proportion in circulation. It is generally accepted that H1 influenza strains tend to cause milder disease than H3 strains [31]–[33]. Pediatric influenza hospitalization was the lowest in 2006. That year, influenza A/New Caledonia/20/99 (H1N1)-like virus accounted for 93.2% of influenza detected after being in circulation at low level in Hong Kong since 1999. The effect on hospitalization also depends on the degree of antigenic drift in the respective strains in circulation, which would affect the level of immunity in the community. In 2008, two drifted H1N1 variants accounting for half of the influenza isolates caused twice the pediatric hospitalization of that seen for A/Brisbane/10/2007(H3N2)-like strain which shared considerable homology with A/Wisconsin/67/2005 (H3N2)-like strain that circulated the year before. In 2011, the A/Victoria/361/2011(H3N2)-like virus appeared in Hong Kong for the first time and contributed to 25% of the isolates. However, it only caused very little hospitalization in young children. This could be because that virus has considerable degree of homology with the A/Perth/16/2009 (H3N2)-like virus that had circulated in the previous 2 years. In addition, the epidemic in 2011 occurred in the summer, when influenza epidemics tend to have lower impact locally than those epidemics that occur in the winter [34].

Influenza hospitalization in the <6 months group deserves some discussion. We had previously suggested that the effect of protection from hospitalization by maternal antibodies could be as high as 25% in infants <6 months [10]. Influenza vaccination uptake amongst pregnant women in Hong Kong were extremely low: 3.9% in 2005–2006 [35] and 6.5% for the H1N1pdm09 vaccine and 4.9% for seasonal influenza vaccine in 2010 [36] so the protection should be from natural infection in women of child-bearing age. Hospitalization for H1N1 was not seen in this youngest group from 2004–2007 when the circulating strain was A/New Caledonia/20/99 (H1N1)-like that had been in the community since 1999. Most women of child-bearing age might have been infected and therefore conferred protection to their young infants. This was not the case when a new strain appeared that the mothers themselves had not encountered. There was significant hospitalization in infants <6 months when 2 new variants A/Solomon Islands/3/2006 (H1N1)-like virus and A/Brisbane/59/2007 (H1N1)-like virus came into circulation in 2008. The significant hospitalization in young infants due to H3N2 for which there was a drift in 2004, 2005 and 2007corroborated with this hypothesis. No hospitalization was documented in <6 months in 2008 for H3N2 despite A/Brisbane/10/2007 (H3N2)-like virus circulated for the first time in Hong Kong, possibly because it shared some homology with the A/Wisconsin/67/2005 (H3N2)-like strain that circulated in 2007. Significant hospitalization in the <6 months group for H1N1pdm09 in the second and third year of circulation in 2010 and 2011 seemed to contradict our hypothesis of maternal antibody protection. However, studies in Hong Kong estimated that the infection attack rate of young adults including women of childbearing age to be much lower than children: 11.8%–13.4% in 20–29 year olds and 4.3–5.8% in 30–39 year olds [20], [21]. There is some evidence on the protection against influenza infection and hospitalization in infants <6 months by maternal influenza vaccination [37], [38]. Our observation provides further support for the protective benefits conveyed by maternal immunity.

There are limitations to the study. Ideally, we should also have information on influenza circulation in children the community. However, the large numbers of specimens available with subtype and strain analysis were a major advantage.

In conclusion, we documented consistently high rates of hospitalization due to influenza A over an 8 year period in Hong Kong. Knowledge of hospitalization due to specific subtypes and proportion of viral circulation provided insight into their impact on disease burden in the pediatric population. Further support for the role of protection from maternal antibodies was provided although further studies to correlate maternal antibodies with disease burden in young infants need to be performed. In light of our present findings, influenza vaccination both in children and pregnant women should be promoted in Hong Kong.
